# Generation of a recombinant rabies Flury LEP virus carrying an additional G gene creates an improved seed virus for inactivated vaccine production

**DOI:** 10.1186/1743-422X-8-454

**Published:** 2011-09-25

**Authors:** Lihong Tao, Jinying Ge, Xijun Wang, Zhiyuan Wen, Hongyue Zhai, Tao Hua, Bolin Zhao, Dongni Kong, Chinglai Yang, Zhigao Bu

**Affiliations:** 1 State Key Laboratory of Veterinary Biotechnology, Harbin Veterinary Research Institute of Chinese Academy of Agricultural Sciences, Harbin 150001, P.R. China; 2 Department of Microbiology and Immunology, Emory University School of Medicine, Rollins Research Center, Atlanta, GA 30322, USA

**Keywords:** Rabies virus, LEP, recombinant, inactivated vaccine

## Abstract

The rabies Flury Low Egg Passage virus (LEP) has been widely used as a seed virus to generate inactive vaccine. Here, we established a reverse genetic system for LEP and generated a recombinant LEP virus (rLEP-G) that carries two identical G genes. This recombinant virus showed similar properties to those of LEP with respect to *in vitro *growth, neurotropism index, and virulence in mice. rLEP-G produced 4.3-fold more G protein than did LEP in BHK-21 cells. The inactivated vaccine generated from rLEP-G induced significantly higher virus neutralization titers in mice and dogs than those produced in response to LEP-derived vaccine. Our results suggest that rLEP-G is an improved seed virus candidate for inactivated rabies virus vaccine manufacture.

## Introduction

Rabies virus (RV) belongs to the genus Lyssavirus of the family Rhabdoviridae, and causes a fatal neurological disease in humans and animals [1] More than 55,000 people die of rabies each year, and about 95% of these deaths occur in Asia and Africa [2]. An estimated 31,000 people die from dog rabies in Asia each year, with most cases occurring in India and China [3,4]. The most cost-effective strategy for preventing rabies in people is to eliminate rabies in dogs via vaccination [5-7]Inactivated rabies vaccine has been shown to be a safe and efficient means to control rabies in dogs. However, the vaccination rate of dogs in many developing countries is low, especially in rural areas, mainly due to low economic development and the high cost of vaccination[8] More efficient and lower cost inactivated vaccine is, therefore, still needed.

The surface glycoprotein (G) of RV is the major antigen responsible for the induction of protective immunity [1] Increasing G protein levels should, therefore, enhance the protective viral neutralization antibody (VNA) response. The rabies Flury low egg passage virus (LEP) has been widely used as a seed virus to generate inactive vaccine for humans and animals because of its high immunogenicity and high growth titer in cell culture [9]. Here, we generated a recombinant LEP virus that carries two identical G genes to increase G protein expression. Growth curves, neurotropism index, virulence, and the G protein expression level of the double-G LEP were tested *in vitro *and *in vivo*. The immunogenicity of the inactivated vaccine derived from this double-G LEP was also evaluated in mice and dogs and compared with that of LEP.

## Materials and Methods

### Viruses and cells

Neuroblastoma (NA) cells of A/J mouse origin were grown in Eagle's minimum essential medium (MEM) supplemented with 10% fetal bovine serum (FBS). Baby hamster kidney (BHK-21) cells were grown in Dulbecco's modified Eagle's MEM (DMEM) supplemented with 10% FBS. The RV LEP (AV2012) was obtained from the China Veterinary Culture Collection Center and propagated in BHK-21 cells. A street virus, GX/09, was isolated from the brain of a dog that died of rabies and was propagated in the brain of adult mice. All viruses were kept in at -70°C before use.

### Plasmids construction

**Figure 1:**
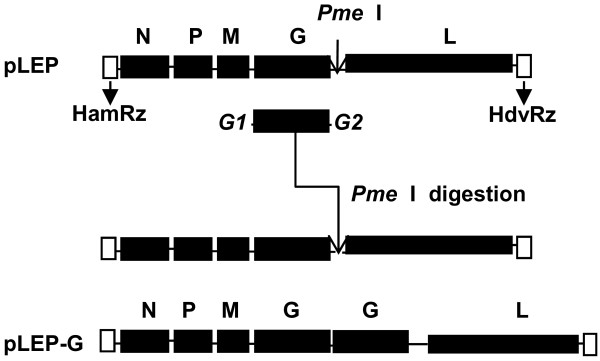
**Schematic representation of recombinant virus containing two homologous G genes**. The pLEP vector was obtained by assembling three overlapping cDNA fragments of the Flury LEP strain into the vector pCI, as described in the Methods. The 1801-bp fragment including the G gene ORF was introduced into the LEP genome through the *Pme *I site in the G-L noncoding region. The hammerhead ribozyme sequence (HamRz) was introduced upstream of the LEP genome, and the hepatitis delta virus ribozyme sequence (HdvRz) was introduced downstream of the viral genome.

Viral RNA was extracted with an RNeasy mini kit according to the manufacturer's instructions (QIAGEN, Valencia, CA). The extracted RNA was subjected to RT-PCR with virus specific primer pairs (Table 1) and high-fidelity *Pfx *DNA polymerase (Invitrogen Corp., Carlsbad, CA) to generate three overlapping PCR fragments (F1, F2, and F3) that encompassed the entire viral genome. The assembled cDNA, containing the hammerhead ribozyme sequence (HamRz), the full-length (11,925-nucleotide) cDNA of the LEP strain genome in the antigenomic orientation, and the hepatitis delta virus ribozyme sequence (HdvRz), was inserted between the *Nhe *I and *Sma *I sites of pCI. A *Pme *I restriction site was introduced into the G-L noncoding region by changing three nucleotide residues at positions 4907 (T to G), 4910 (G to T) and 4912 (C to A) by using a site-directed mutagenesis system (Invitrogen) with the primers shown in Table 1. The resultant plasmid was designated as pLEP. The cDNA of 1,801 nucleotides including the open reading frame of the G gene was amplified from pLEP by the primer pair shown in Table 1. The fragment was introduced into the LEP genome through the *Pme *I site. The resultant plasmid was designated as pLEP-G (Figure 1). The open reading frames (ORFs) of the N, P, and L genes were PCR-amplified from pLEP-G with the primers shown in Table 1 for the construction of the N, P, and L expression plasmids. The amplified N, P, and L genes were inserted between the *EcoR *I and *Kpn *I sites in the plasmid pCAGGS and were designated as pCA-N, pCA-P, and pCA-L, respectively. The assembled full-length cDNA clone and the helper plasmids were sequenced in their entirety to ensure that no undesirable mutations had been introduced.

**Table 1:** Primers used to construct the full-length cDNA clone and helper plasmids for Flury LEP

Purpose	Name	Primer (5'-3')^a^
For F1 amplification	F1-F	TGC**GCTAGC***TGTTAAGCGTCTGATGAGTCCGTGAGGACGAAACTATAGGAAAGGAATTCCTATAGTC*ACGCTTAACAACAAAACCAAAGAA^b^
	F1-R	GGC**ACGCGT**ACTCCACATAACTTGAGTTTGC

For F2 amplification	F2-F	AGGCCTGTATAAGTCTTTAAAGGGAGCA
	F2-R	ATCGGGGTTCCCGGCCTCTTGACACAAC

For F3 amplification	F3-F	TAT**GCTAGC**TCTGGTTAAGCTCCCACGAATC
	F3-R	CGAT**CCCGGG**ccccgcgggggcccctcccttagccatccgagtggacgaacgtcctccttcggatgcccaggtcggaccgcgaggaggtggagatgccatgccgacccACGCTTAACAAATAAACAAT^c^

For Pme I mutation	Pmu-F	GACTTGAAGTTTAAACAGGATGACCGGCC^d^
	Pmu-R	GGCCGGTCATCCTGTTTAAACTTCAAGTC

For G gene amplification	G1	ATGC**GTTTAAAC**AAGTTTATCACTTGTTTACCTCT
	G2	GCAT**GTTTAAAC**ACTTGAAGTGTCAAAAGGATGA

For N gene amplification	HN-F	GGC**GAATTC**ATGGATGCCGACAAGATTGT
	HN-R	CCG**GGTACC**TTATGAGTCACTCGAATACG

For L gene amplification	HL-F	GGC**GAATTC**ATGCTGGATCCGGGAGAGGTTT
	HL-R	CCG**GGTACC**TTACAAACAACTGTAGTCTA

For LEP-G confirmation	G3	ATGCTTTCTCTTGAATGTGG
	G4	GGGTTTGGAAAAGCATATAC

^a ^Restriction enzyme sites are shown in boldface. ^b ^The HamRz sequence is shown in italics. ^c ^The HdvRz sequence is shown in lowercase. ^d ^The nucleotides that were changed are underlined.

### Virus rescue

BHK-21 cells were grown overnight to 80% confluence in 6-well plates in DMEM supplemented with 10% FBS. Cells were transfected with 4.0 μg of the full-length plasmid pLEP-G, 2 μg of pCA-N, 1 μg of pCA-P, and 1 μg of pCA-L by using Lipofectamine™ 2000 (Invitrogen) according to the manufacturer's protocol. After 4-6 h, the transfection medium was replaced with fresh medium. After 3 days, supernatants were transferred onto BHK-21cells and incubated for a further 3 days. Rescued viruses were examined by using an indirect fluorescence assay (IFA) with mouse anti-RV serum and FITC-conjugated goat anti-mouse IgG (Sigma). Supernatants from virus-positive cultures were collected to propagate virus stocks in BHK-21 cells. Sequences of recovered viruses were confirmed by sequencing entire viral genomes. To confirm whether the recovered rabies virus was derived from the cloned pLEP-G, RT-PCR was performed with the primers shown in Table 1, positive-sense G4 and negative-sense G3 primers at nucleotide positions 4,302 to 4,321 and 3,613 to 3,632 (based on the genomic nucleotide number of the LEP strain), respectively. The fragment was further digested with *Pme *I, and the supernatants from virus-positive cultures were used to produce virus stocks in BHK-21 cells. The rescued virus generated from the full-length plasmid pLEP-G was designated as rLEP-G.

### Multistep growth assays

Confluent BHK-21 or NA cell monolayers grown in 6-well plates were infected with LEP or rLEP-G at a multiplicity of infection (MOI) of 0.01. After incubation for 1 h at 37°C, the inoculums were removed and the cells were washed twice with PBS. BHK-21 and NA cells were replenished with DMEM containing 2% FBS or with MEM containing 0.2% FBS, respectively, and incubated at 37°C. Culture supernatant (100 μl) was harvested at 0, 1, 2, 3, 4, and 5 days post-inoculation for virus titration.

### Virus titration

Monolayers of NA or BHK-21 cells in 24-well plates were infected with 10-fold dilutions of virus suspension and incubated at 37°C. At 48 h post-infection, an IFA test was performed. Foci were counted under a fluorescence microscope and calculated as focus forming unit/ml (FFU/ml).

The *in vitro *neurotropism index was expressed as the logarithm of the titer of virus stock in NA cells minus the logarithm of the titer of the same stock in BHK-21 cells [10]. The virus titers for neurotropism index calculation were based on the titration results of virus stock harvested at 72 h post infection.

### Western blotting

BHK-21 cells grown in 6-well plates were infected with LEP or rLEP-G at an MOI of 1 and incubated for 48 h. G gene expression was confirmed by using western blotting as described previously [11]. Briefly, cell extracts were subjected to 10% polyacrylamide gel electrophoresis (SDS-PAGE), and then blotted to nitrocellulose membrane. The membrane was incubated with a mixture of mouse anti-G protein polyclonal antiserum and anti-β-actin monoclonal antibody (Santa Cruz Biotechnology), as the first antibody, and then with IRDye700-conjugated anti-mouse IgG (Rockland, Gilbertsville, PA) as the second antibody. The mouse anti-G protein polyclonal antiserum was generated by immunization with a recombinant Newcastle disease virus that expresses the RV glycoprotein and was developed in our lab. Signal intensities were analyzed by using the Odyssey infrared image system (LiCor). The densitometry of each band was quantified with Photoshop CS2. The G/β-actin ratio was calculated by dividing the densitometry of the G protein band by that of the β-actin protein band.

### Infection of mice

The virulence of LEP and rLEP-G for adult mice was measured in 6-week-old female Balb/c mice (Vital River, Beijing). To determine the 50% lethal dose (LD_50_) of each virus, groups of five adult mice were inoculated intracerebrally (*i.c*.) with 30 μl or intramuscularly (*i.m*.) with 100 μl of serial 10-fold dilutions of LEP or rLEP-G. After infection, mice were observed for 21 days for clinical signs or death. The LD_50 _of each virus was calculated by using the method of Reed and Muench [12].

### Preparation of inactivated vaccines

BHK-21 cells grown in 75 cm^2 ^flask were infected with LEP or rLEP-G at an MOI of 0.01 and incubated at 37°C. At 96 h post-infection, cells were suspended in the supernatant by using a rubber policeman. The suspension was submitted to three cycles of freezing and thawing (-20°C/37°C, 5 min each) and then subjected to ultrasonic sound to increase the cell extraction dissolution. After centrifuge, supernatants were collected and stored in -70°C. The infectious viruses in the supernatant were titrated in BHK cells. To prepare inactivated vaccines, the supernatant was incubated at 37°C for 2 h in the presence of 0.03% β-propiolactone. Then, three volumes of inactivated supernatant preparation (non-dilution, 20-f old dilution, and 80-fold dilution) were mixed with one volume of Rehydraphos AlPO_4 _adjuvant (Reheis, Berkeley Heights, NJ), and stored at 4°C for vaccination.

### Immunization of Balb/c mice and dogs

Groups of ten 4-week-old female Balb/c mice were inoculated *i.m. *in the gastrocnemius muscle with 100 μl of 20- or 80-fold dilutions of inactivated vaccine preparations. Groups of six 3-month-old Beagles were inoculated *i.m. *with 1 ml of inactivated vaccine preparations.

### VNA assay

Three weeks after immunization, mice were bled from the retro-orbital sinus under isoflurane inhalation anesthesia and Beagles were bled from the vein of the front leg. Sera were tested for neutralizing antibodies (VNA) by using the rapid fluorescent focus inhibition test (RFFIT) [13]. Neutralization titers, defined as the highest serum dilution that neutralizes 50% of the challenge virus, were normalized to international units (IU) according to the World Health Organization anti-RV antibody standard.

## Results

### Recovery of rLEP-G from cloned cDNA

**Figure 2:**
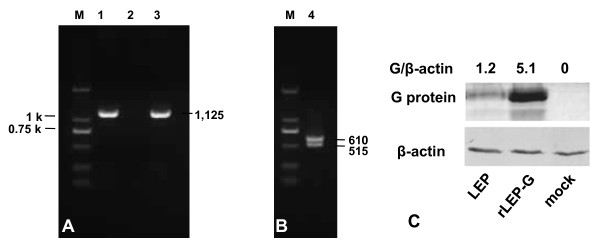
**Confirmation of recovery of the rLEP-G from cloned cDNA**. (A) RT-PCR, with the G3 and G4 primers, detected the genome RNA from rLEP-G after two passages in BHK-21 cells (lane 1), LEP (lane 2), and rLEP-G after 10 passages in BHK-21 cells (lane 3). (B) The RT-PCR amplified cDNA fragment was digested with *Pme *I. M, molecular weight marker. (C) Western blot analysis of G protein expression in BHK-21 cells infected with viruses. Cells were collected at 96 h post-infection with LEP or rLEP-G and subjected to Western blot analysis as described in the Methods. The G/β-actin ratio was calculated by dividing the densitometry of the G protein band by that of the β-actin protein band.

The full-length genome plasmids of recombinant LEP carrying two identical G genes were assembled and used to rescue recombinant virus. The resulting virus was designated as rLEP-G. To exclude the possibility of contamination with LEP, a cDNA fragment covering the G-G noncoding region was amplified from the genomic RNA of the rescued recombinant virus and LEP, respectively, by using RT-PCR with a set of primers, G3 and G4. The amplified cDNA fragments were digested with *Pme *I. A fragment with the expected size of 1,125 bp was amplified from the genomic RNA of rLEP-G, whereas RT-PCR with the genomic RNA of LEP did not amplify any detectable product (Figure 2A). The amplified cDNA fragment was digested with *Pme *I, and 610-bp and 515-bp bands were observed (Figure 2B). Sequence analysis further confirmed that the virus was rescued from cloned pLEP-G. RV G protein expression by rLEP-G in BHK-21 cells was compared with that of LEP by using Western blot analysis. G protein was expressed at a significantly higher level in rLEP-G-infected cells than in LEP-infected cells (Figure 2C). In LEP-infected cells, the G/β-actin ratio was 1.2, whereas in rLEP-G-infected cells the G/β-actin ratio was 5.1, indicating an approximate 4.3-fold increase in G protein levels in rLEP-G-infected cells due to expression from the additional G gene.

### Growth properties of rLEP-G *in vitro*

**Figure 3:**
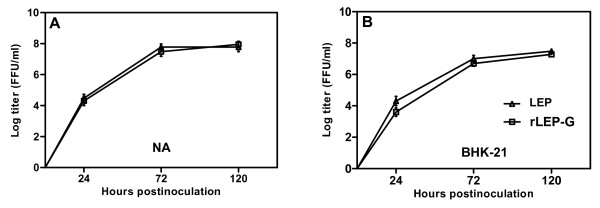
**Multistep growth curves for LEP and rLEP-G**. NA cells (A) and BHK-21 Cells (B) were infected at an MOI of 0.01. The infected cell culture supernatants were harvested at different time post-inoculation, and virus titers were determined in triplicate in BHK-21 cells.

To examine whether insertion an identical G gene into the genome of the LEP strain would affect the growth properties of the virus *in vitro*, we compared the growth of rLEP-G and LEP in both neuronal NA cells and non-neuronal BHK-21 cells. The multistep growth curves of rLEP-G (the third passage generation from BHK-21 cells) were compared with that of LEP in NA cells. There were no significant differences in growth patterns in both cell lines (Figure 3A, B) between rLEP-G and LEP. Furthermore, LEP and rLEP-G showed similar *in vitro *neurotropism indices of 0.84 and 0.85, respectively, which indicates that the additional G gene did not affect the *in vitro *neurotropism of the LEP strain.

**Figure 4:**
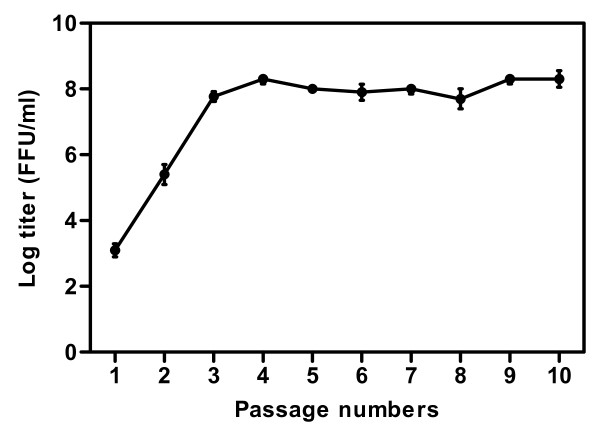
**Virus titers of rLEP-G serially propagated in BHK-21 cells**. BHK-21 cells were infected with rLEP-G at an MOI of 0.01. The infected cell culture supernatants from each passage were harvested at 96 h post-inoculation, and virus titers were determined in triplicate in BHK-21 cells.

To examine the genome stability of rLEP-G in cell culture, the virus was serially passaged in BHK-21 cells 10 times. RT-PCR analysis revealed that the additional G gene in the rLEP-G genome was stable for at least 10 passages in BHK-21 cells. The titer of rLEP-G also remained high at approximately 1 × 10^8 ^FFU/ml, from the third to the tenth passage (Figure 4).

### Pathogenicity of rLEP-G in adult mice

**Figure 5:**
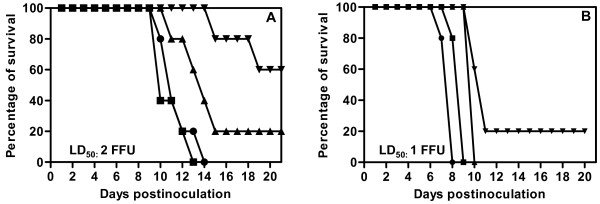
**Pathogenicity of LEP and rLEP-G in adult mice**. Six-week-old Balb/c mice were inoculated intracerebrally with LEP (A) or rLEP-G (B) in a 30 μl volume at doses of 10^0 ^to 10^3 ^FFU (black inverted triangle:10^0 ^FFU; black triangle: 10^1 ^FFU; black square: 10^2 ^FFU; black dot: 10^3 ^FFU). After infection, mice were observed for 21 days for clinical signs or death. The MLD_50 _of each virus was calculated by using the method of Reed and Muench.

The G protein plays an important role in the pathogenicity of RV. Therefore, we needed to investigate whether insertion of an additional G gene would affect virulence. Accordingly, adult mice were inoculated with rLEP-G and LEP, respectively. The results showed that both rLEP-G and LEP caused neurological symptoms, such as paralysis and hyperactivity, and killed mice after i.c. inoculation. The LD_50 _of rLEP-G following i.c. inoculation was 2 FFU, which was 2-fold higher than that of LEP (Figure 5). Peripheral pathogenicity was also examined following i.m. inoculation. All mice survived i.m. inoculation with 10^6 ^FFU of rLEP-G, whereas, one of the five mice inoculated i.m. with the same dose of LEP died. This result indicates that the *in vivo *neuro-invasiveness and peripheral pathogenicity of LEP did not increase after insertion of the additional G gene.

### Induction of VNA in mice and dogs

**Figure 6:**
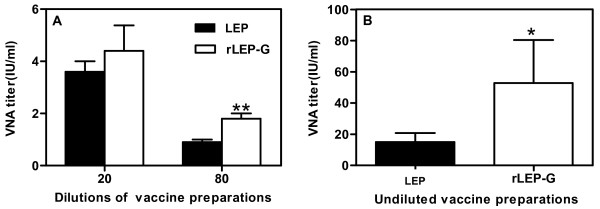
**VNA titers in mice and dogs immunized with inactivated LEP or rLEP-G vaccine preparations**. (A) Groups of 10 mice were immunized with 20- or 80-fold dilutions of inactivated vaccine preparations of LEP or rLEP-G. (B) Groups of 6 dogs were immunized with 1 ml of inactivated vaccine preparations of LEP or rLEP-G. Blood samples were collected at 3 weeks post-vaccination and subjected to the VNA titration test. VNA were determined by using the rapid fluorescence inhibition test. Titers were normalized to international units by using the World Health Organization standard. **, *P *< 0.01; *, *P *< 0.05.

VNA is mainly induced by viral antigen G protein and plays a key role in immune protection from rabies. To investigate whether increased expression of G in the seed virus improves the VNA response of inactivated rabies vaccine, two inactivated vaccines were respectively preparations from LEP and rLEP-G infected cell cultures as described in materials and methods. The virus titers in supernatant of LEP and rLEP-G infected cell cultures were 10^7.4 ^FFU/ml and 10^7.3 ^FFU/ml respectively. The preparations were then tested in adult mice by using *i.m. *inoculation with. There were no significant differences in VNA response between the mice that received 0.1 ml of 20-fold diluted LEP vaccine preparation (containing 5.2 log_10 _FFU/0.1 ml) and those that received 0.1 ml of 20-fold diluted LEP-G vaccine preparation (for LEP and 5.1 log_10 _FFU/0.1 ml for rLEP-G). However, the VNA of mice immunized with an 80-fold diluted rLEP-G vaccine preparation (containing 4.5 log_10 _FFU/0.1 ml) was significantly higher than that of mice immunized with a similarly diluted LEP vaccine preparation (containing 4.4 log_10 _FFU/0.1 ml) (P < 0.01) (Figure 6A). In dogs, 1 ml of rLEP-G vaccine preparation (containing 7.3 log_10 _FFU/ml) induced a VNA mean titer of 54 IU, which was significantly higher than mean titer of 15 IU that induced by 1 ml of LEP inactivated vaccine (containing 7.2 log_10 _FFU/ml) (P < 0.05) (Figure 6B). These results indicate that the rLEP-G strain is a more immunogenic seed virus for use in the development of inactive vaccines against rabies.

## Discussion

Mass vaccination of dogs has proven to be an effective way to control rabies in this species. However, current live attenuated vaccines can have safety issues [14,15], and the use of current inactivated vaccines is limited in developing countries because of high manufacturing costs. Increasing G protein expression in RV seed viruses is hypothesized to improve rabies inactivated vaccines not only via immunogenicity but also through increased manufacturing productivity. Previous studies have demonstrated the feasibility of introducing an additional G gene into the RV genome [16-18]. By using reverse genetics technology, Faber et al. generated a double-G gene recombinant RV (SPBNGA-GA). This double-G virus showed a 1.6-fold increase in G protein expression in cell culture relative to that of its single-G gene parent virus (SPBNGA). Inoculation with SPBNGA-GA live virus provided more efficient protective immunity than did the SPBNGA strain in mice. However, the utility of SPBNGA-GA as a seed virus for inactivated vaccine production has not been evaluated further [18]. In another study, Hosokawa-Muto et al. generated a double-G gene recombinant RV RC-HL. This virus showed a 1.5-fold increase in G protein expression in cell culture compared with that induced in the single-G gene parent virus RC-HL[16]. When the recombinant double-G gene RC-HL and wild-type RC-HL were inactivated and used to vaccinated mice, no significant difference in RV neutralization antibody responses was observed.

Here, we generated a recombinant LEP virus carrying an additional G gene, rLEP-G. This recombinant virus produced strikingly higher levels of G protein in cell culture and showed similar *in vitro *growth properties and bio-safety characteristics. Inactivated vaccine prepared from rLEP-G induced significant higher RV VNA titers in mice and dogs than those induced by LEP-derived vaccine, indicating that rLEP-G represents an improved seed virus candidate for inactivated RV vaccine manufacture.

Since G protein is a major contributor to the pathogenicity of rabies virus [19-22], we evaluated whether this increase in G protein expression affected the virulence of the rLEP-G virus. We found that the insertion of this additional G gene results in increase of G protein expression but not in increase of the neuro-invasiveness or peripheral pathogenicity of the virus in adult mice. In other studies, introduction of one or two additional G genes into the genome of rabies virus resulted in a higher level of G protein expression and attenuate pathogenicity in adult mice [17,18,23]. As the pathogenicity of a particular RV strain correlates inversely with its ability to trigger apoptosis in neuron cells[23], one possible explanation for this discrepancy is that some lethal viruses, such as LEP and rLEP-G, rarely induced apoptosis in neuron cells. In fact, our previous data had shown that there was no clear correlation between apoptosis induction and the viral replication titers or glycoprotein expression level among LEP and its relative mutants or chimerical viruses[19].

## Competing interests

The authors declare that they have no competing interests.

## Authors' contributions

LT, HZ and TH carried out the plasmid construct cloning, rescued and characterized the recombinant virus. LT, JG, XW and ZW carried out the immunization assay and data analysis. BZ and DK participated in the animal immunization. ZB designed the whole study, provided general supervision and prepared the manuscript. CY participated in experiment design and helped preparing the manuscript. All authors have read and approved the submitted manuscript.
